# Privacy and personalisation: predicting Parkinson’s disease severity from real-world gait with federated learning

**DOI:** 10.3389/fnagi.2026.1766599

**Published:** 2026-03-09

**Authors:** Chloe Hinchliffe, Hugo Hiden, Lisa Alcock, Rachael A. Lawson, Alison J. Yarnall, Lynn Rochester, Silvia Del Din, Paul Watson

**Affiliations:** 1 Translational and Clinical Research Institute, Faculty of Medical Sciences, Newcastle University, Newcastle upon Tyne, United Kingdom; 2 School of Computing, Newcastle University, Newcastle upon Tyne, United Kingdom; 3 National Institute for Health and Care Research (NIHR) Newcastle Biomedical Research Centre (BRC), Newcastle University and The Newcastle upon Tyne Hospitals NHS Foundation Trust, Newcastle upon Tyne, United Kingdom; 4 The Newcastle upon Tyne Hospitals NHS Foundation Trust, Newcastle upon Tyne, United Kingdom

**Keywords:** digital wearables, edge device, explainable AI, federated learning, gait, IMUs, internet of things (IoT), Parkinson’s disease

## Abstract

**Introduction:**

Cloud-based artificial intelligence (AI) combined with smart-health technology presents a powerful tool to passively monitor disease severity. However, current methods raise privacy concerns as they require transmitting patient data to the cloud. A potential solution is Federated Learning (FL), which only shares the weights of locally trained neural networks (NNs) instead of user data. Here, we simulated an FL system to demonstrate its application for evaluating Parkinson’s disease (PD) severity in a smart-home scenario.

**Methods:**

Retrospective data including 89 people with PD wore an accelerometer on the lower-back at home for 7 days at 18-month intervals over 6 years. Patient characteristics (age, sex, and body mass index) and clinical measures of PD were additionally collected, including the Movement Disorder Society Unified Parkinson’s Disease Rating Scale (MDS-UPDRS)-Part III. Real-world daily gait measures along with these patient characteristics were used to predict the MDS-UPDRS-III score. For FL, a local model was trained for each participant, and a global model (an aggregation of these local models) was tested on unseen participants.

**Results:**

The performance of a simulated FL system was compared with that of a traditional Machine Learning (ML) approach in which patient data were shared. The traditional ML approach had a mean absolute error (MAE) of 10.43. The global FL model had a similar MAE of 10.22 but was underfitted, and the mean MAE of the local, personalised models was 4.83. Shapley Additive exPlanations (SHAP) analysis showed that while the participants’ age and sex were very important in traditional ML, this was not the case for the local FL models, leading to a decrease in global model performance. Here, we show that reserving a small number of participants from the system and including them in training data for all local models restored the importance of these features and improved global FL performance (MAE = 9.26) but reduced local performance (MAE = 6.83).

**Conclusion:**

This exploratory study shows that our proposed approach enables FL to achieve similar accuracy to traditional Machine Learning without sharing any patient data but with costs to the local performance, leading towards a smart-home system that prioritises personalisation and patient privacy.

## 1 Introduction

Parkinson’s disease (PD) is a neurodegenerative disease with an estimated global prevalence of 8.5 million in 2019 (World Health Organization, 2023) and the global prevalence is set to rise, even after correcting for age related factors (Bloem et al., 2021). PD is characterised by motor symptoms such as bradykinesia (slow movement), tremor, and gait impairments, and includes non-motor features such as cognitive impairment, mood disorders, and sleep disturbances (Bloem et al., 2021; World Health Organization, 2023). PD is progressive and incurable, but symptomatic treatments are available. Current treatment plans focus on the management of these symptoms through medication and non-pharmacological interventions (Bloem et al., 2021). The severity of PD symptoms is assessed using clinical measures such as Movement Disorder Society Unified Parkinson’s Disease Rating Scale (MDS-UPDRS) which is comprised of four parts: non-motor experiences of daily living, motor experiences of daily living, motor examination, and motor complications, and combines assessment by a trained professional with self-reporting (Goetz et al., 2008).

For a clinical trial testing an intervention intended to improve PD symptoms, changes in clinical measures such as the MDS-UPDRS score would need to be monitored to assess intervention efficacy. However, these clinical measures are relatively subjective, have variable inter-rater reliability, can contain substantial within-subject error variance, and do not account for fluctuating PD symptoms (Evers et al., 2019; Hendricks and Khasawneh, 2021). To address these challenges in clinical research, TORUS aims to develop a novel platform of sensing technologies to be deployed within a patient’s home. This system will supplement current clinical scores, such as MDS-UPDRS, by extracting clinically important mobility features from cameras in the home and a wrist-worn wearable. This system will also combine smart-home and machine learning technologies to build a system that continuously and passively monitors changes in PD symptoms.

To train a neural network (NN) that can predict changes in PD symptoms using conventional Machine Learning (ML), this smart home-based system would require the transmission of sensitive patient information from their houses to a central location where the ML takes place. This presents serious privacy concerns for participants, and many potential beneficiaries would be reluctant to have personal data such as video footage, wearable recordings, and data from other sources transmitted outside of their home. To address these concerns, we are exploring the use of Federated Learning (FL) (McMahan and Ramage, 2017). Here, instead of only one NN built by ML from data sent to a central location, NNs are built for each individual participant within their own homes—these are the *local models*—and only information about these local models, in lieu of the patients’ data, are transmitted to a central location. This local model information is aggregated together into one NN: the *global model*. This means that this federated system can allow an NN in a central location to learn from many individual participants without sharing any patient data, therefore presenting an interesting opportunity to deploy artificial intelligence (AI) in the home whilst protecting patients’ privacy.

**Figure 1:**
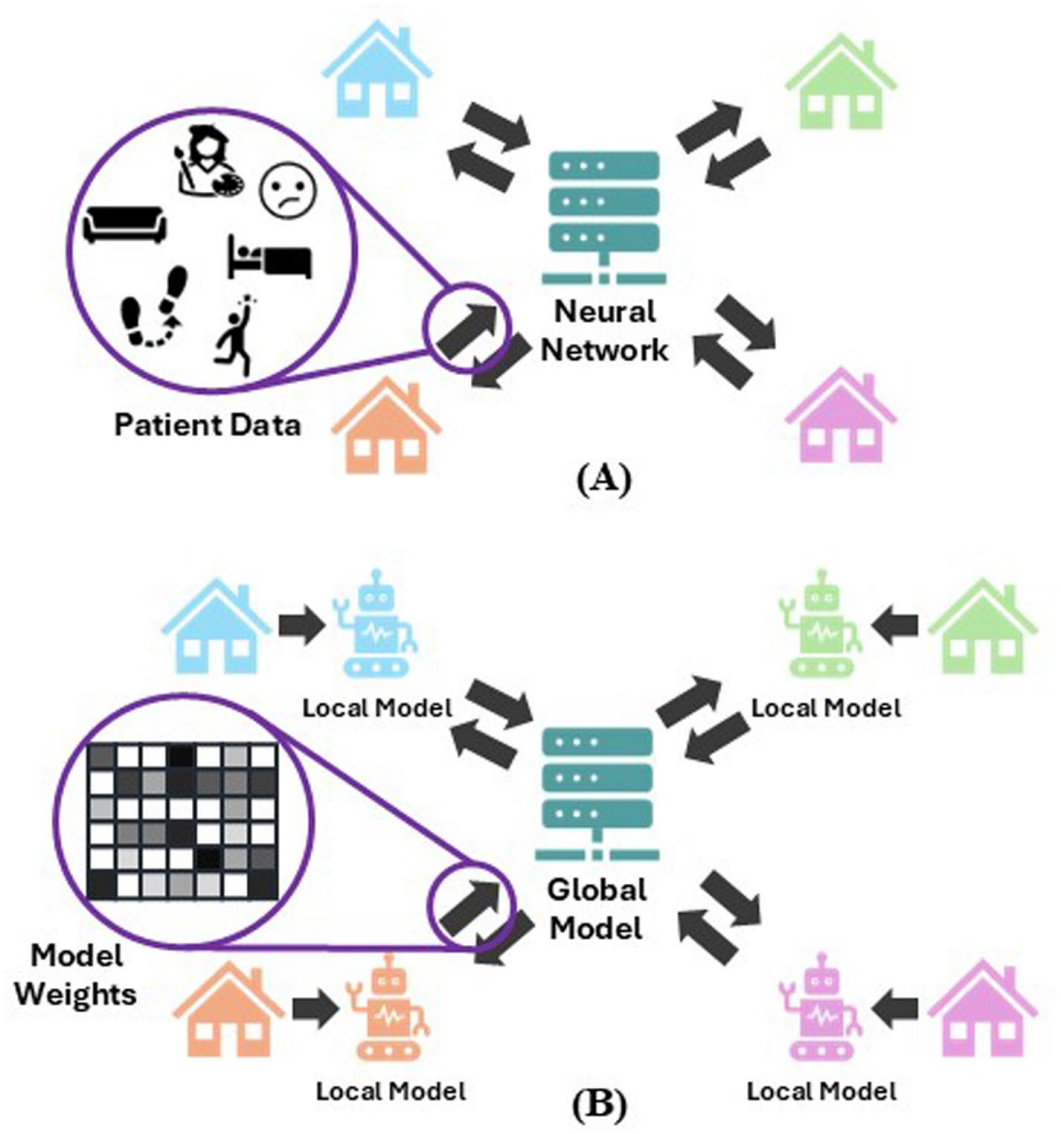
**(A)** Visualisation of a traditional smart home set up, where patient data is collected within the home and transmitted to a central location where the traditional neural network is built. **(B)** Visualisation of a federated system, where local models are built within the patients’ homes and the local models’ weights are transmitted to a central location instead of patient data. The local models are aggregated into the global model and redistributed to the patients’ homes for further training. This is repeated in a cyclic process for a set number of iterations.

In a federated system, *clients* are the devices that manage the local models, and a *server* manages the global model as well as communication with the clients. The model information transmitted to the central server are the model weights. These are numerical values that determine the strength of the relationship between two neurons in the network and are the parameters that are “learned” during training. Interpreting information about the original training data from model weights, or indeed inferring any meaningful information from model weights, is incredibly difficult, leading to these sorts of models being known as “black box” models. Therefore, transmitting this information is a lot safer than simply transmitting encrypted patient data, since even the engineers who built the system would not be able to discern anything meaningful about the participants. These learned local model weights are aggregated by the server into the global model using techniques such as *FedAvg* (Brendan McMahan et al., 2016), where the model weights are averaged with the mean weighted by the number of samples in the local data set. The global model will then be sent out to the clients to be evaluated on local test data and then updated with further training on the local training data. These updated model weights are then sent back to the server to be aggregated again. This process is repeated for a set number of *rounds*, with the expectation that at the end of these rounds the local models in the system will be accurate for making predictions for their local data and the global model will be accurate for making predictions on new, unseen participants. Another advantage of a federated system in a smart home scenario is each participant would have their own NN, meaning this model can be tailored to their specific data, giving each participant a personalised model. Figure 1 show visualisations of a federated system (B), compared to a traditional machine learning network (A).

The most common use of Federated Learning in healthcare research has been where hospital sites were used as clients, allowing machine learning models to learn from multiple centres without sharing their imaging, sensor, or electronic health record (EHR) data (Zhang et al., 2024). Federated learning has also been previously explored in PD research. Studies have used data including motor symptoms (Chen et al., 2020, 2023; Jorge et al., 2022, 2024; Guan et al., 2024; Soumma et al., 2024; Tanim et al., 2024), speech (Joshi et al., 2023; Arasteh et al., 2023; Sarlas et al., 2023; Ducange et al., 2024), imaging (Dipro et al., 2022; Souza et al., 2024), questionnaires (Reyes et al., 2021), genetic information (Danek et al., 2024), and facial expressions (Pang et al., 2025). These studies have aimed to identify PD participants from controls (Joshi et al., 2023; Chen et al., 2020; Dipro et al., 2022; Arasteh et al., 2023; Sarlas et al., 2023; Danek et al., 2024; Souza et al., 2024; Tanim et al., 2024; Pang et al., 2025), to predict PD symptom severity (Reyes et al., 2021; Chen et al., 2023; Ducange et al., 2024; Guan et al., 2024; Jorge et al., 2024), or to predict freezing of gait (Jorge et al., 2022; Soumma et al., 2024), and partitioned their data by grouping participants into institutions for the clients (Joshi et al., 2023; Chen et al., 2020, 2023; Reyes et al., 2021; Dipro et al., 2022; Arasteh et al., 2023; Sarlas et al., 2023; Danek et al., 2024; Ducange et al., 2024; Guan et al., 2024; Souza et al., 2024; Tanim et al., 2024; Pang et al., 2025) or using an individual participant for each client – in computer science this is called an edge device case (Jorge et al., 2022, 2024; Soumma et al., 2024).

Three studies explored edge device cases aimed to detect freezing of gait (FoG) (Jorge et al., 2022; Soumma et al., 2024) and tremor (Jorge et al., 2024). All three used a type of NN: two used FedAvg to aggregate the local models (Jorge et al., 2022, 2024), while one study does not report the aggregation strategy (Soumma et al., 2024). All saw reduced performance in their global federated NN compared to traditional NN: convolutional NN (CNN) balanced accuracy of 81% reduced to 76% (Jorge et al., 2022); CNN accuracy of 87.23% reduced to 86.98% (Soumma et al., 2024); and CNN balanced accuracy of 73.5% reduced to 63.2% and CNN-long short term memory (LSTM) balanced accuracy of 72.1% reduced to 66.2% (Jorge et al., 2024). Notably, all three studies used small samples (10 PD (Jorge et al., 2022), 62 PD (Soumma et al., 2024), and 27 PD (Jorge et al., 2024) participants) and all data were collected in a lab setting.

Overall, the majority of previous studies exploring FL in PD grouped participants into two or more hospital sites, and the limited number exploring edge device cases used small, lab-based data sets and did not report the accuracy of the local models.

Our work will address this gap and include personalisation for the local models and explainable AI analysis to explore the impacts of FL on feature importance. It will assess the capabilities and challenges of using wearable devices to extract measures of mobility and evaluate the severity of PD symptoms in an edge device case FL system. We do this through analysis of a simulated FL system and compare performance to a traditional machine learning approach using a pre-existing dataset comprised of real-world digital gait data from people with PD.

## 2 Materials and methods

### 2.1 Data

We conducted analysis on an existing dataset: Incidence of Cognitive Impairment in Cohorts with Longitudinal Evaluation - PD (ICICLE-PD) (Yarnall et al., 2014; Lawson et al., 2021) and the nested ICICLE-GAIT (Lord et al., 2013). Participants were recently diagnosed with PD from the community and hospital outpatient clinics in Newcastle-upon-Tyne, UK. As a part of ICICLE-GAIT, 121 participants with PD completed five assessments 18 months apart, however the initial baseline visit was not used in the current analysis. Demographic information including date of birth and gender were collected at baseline, and participants were assessed in a lab setting using MDS-UPDRS and Hoehn and Yahr staging at each timepoint. In addition to the clinical assessments, the participants wore a triaxial accelerometer on the lower-back in the real world for up to seven continuous days. The triaxial accelerometer used was the Axivity AX3 device (Axivity Product, 2025) which had a sampling rate of 100 Hz and a range of ±8 g (1 g is equivalent to 9.81 m/s^2^).

The study was approved by the Newcastle and North Tyneside 1 Research Ethics Committee and performed according to the Declaration of Helsinki. All participants provided written informed consent. PD participants were diagnosed by a neurologist specialising in movement disorders. Exclusion criteria for the ICICLE-PD and ICICLE-GAIT studies included: significant cognitive impairment or meeting Diagnostic and Statistical Manual of Mental Disorders, Fourth Edition (DSM-IV) criteria for dementia; insufficient working knowledge of English; and lacking capacity to give informed consent. Additional criteria for the current analysis excluded participants without a single visit with an MDS-UPDRS Part III score and at least 24 h of corresponding device wear-time, since they did not have sufficient data for the current analysis.

### 2.2 Patient and public involvement

Patients and/or the public were not involved in the study design, conduct, or reporting of this manuscript.

### 2.3 Patient characteristics and real-world daily gait measures

For this analysis, 88 daily digital gait measures were extracted from the data collected by the lower-back accelerometer. The methods to extract these measures have been described in our previous work (Hinchliffe et al., 2024) and used algorithms (McCamley et al., 2012) that have been validated in PD (Del Din et al., 2016). This approach estimated the walking bouts (periods of continuous walking with a minimum of three consecutive steps), from which the following real-world behaviours and gait characteristics of the steps were estimated on a daily level:

• Macro characteristics:
 o *Amount:* Number of walking bouts, total step count, mean step count, total walk time (s).
 o *Pattern:* mean walk time (s).
 o Var*iability:* standard deviation (SD) of walk time (s), SD of step count.
• Micro characteristics:
 o *Pace:* mean step length (m), mean step velocity (m/s), SD of swing time (s).
 o *Rhythm:* mean step time (s), mean stance time (s), mean swing time (s).
 o *Variability:* SD of step time (s), SD of stance time (s), SD of step velocity (m/s), SD of step length (m).
 o *Asymmetry:* asymmetry of step time (s), asymmetry of swing time (s), asymmetry of stance time (s).
 o *Postural control:* asymmetry of step length (m).

Here, asymmetry is the absolute difference between the right and left feet. These daily gait measures were extracted from all identified walking bouts, as well as additionally stratified short (10–30s), moderate (30–60s), and long (>60s) walking bouts. These gait measures were used to train and test the models, along with participant characteristics—age, sex, and body mass index (BMI)—giving 91 parameters to input to the models. If the participant’s BMI was not recorded for a visit, their BMI was taken as the BMI at the most recently recorded value.

### 2.4 Traditional machine learning

Machine Learning models were built to predict PD motor symptom severity using as inputs these daily gait measures and patient characteristics. The PD motor symptom severity was estimated using the MDS-UPDRS Part III, which ranges from 0 to 132 and minimal meaningful change has been reported as 2.3 to 2.7 points, a moderate change as 4.5 to 6.7 points, and a large change as 10.7 to 10.8 points (Shulman et al., 2010). Since the MDS-UPDRS Part III was only collected once for each visit, this score was set as the label for each of the 7 days for that visit. While there are limitations associated with this approach, which will be expanded upon in the Discussion section, it was decided that this was appropriate with these available data.

As a control approach, we performed traditional machine learning with a fully connected NN. The data were split into training and test sets using participant-wise 10-fold cross validation, meaning 10% of participants were used as test data. For each fold, a further 10% of participants were excluded from training as validation data for the NN, leaving 80% of participants for training. Missing values were imputed with the median of the training data and scaled using ScikitLearn’s StandardScaler (Pedregosa et al., 2011). The targets for this analysis were the MDS-UPDRS Part III scores and, to reflect the output of the sigmoid function used by the Output Layer, were scaled to 0–1 for model deployment and scaled back for interpretation of performance.

**Figure 2:**
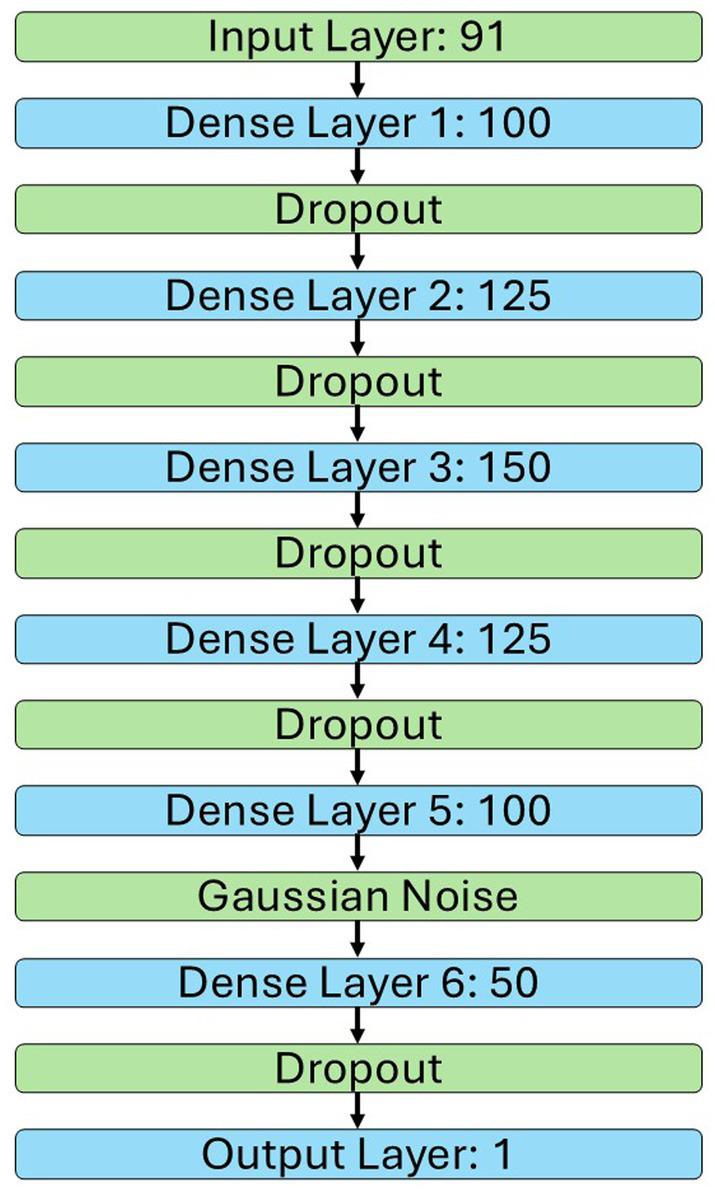
Architecture of the NN. Green represents layers with non-trainable parameters and blue represents layers with trainable parameters.

The architecture for the NN is shown in Figure 2. This model had 77,301 model weights, used an Adam optimiser, and the loss was calculated with mean squared error (MSE). All dropout layers were set at 50%, the SD for the Gaussian noise layer was 0.6. The activation function was ReLU for all layers, except the Output Layer which used a Sigmoid function. Early stopping, with a patience of 20 and maximum epochs of 150, was used to roll back to the weights with the lowest mean absolute error (MAE) for the validation data. These hyperparameters were selected based on initial experimentation. All analyses were done in Python v3.10.13 with TensorFlow v2.10.0 (Abadi et al., 2015) on a Dell Precision 3,660 tower with a 12th Gen Intel Core i3 processor, featuring 12 MB cache and 8 cores.

### 2.5 Federated learning

The Federated Learning simulation replicated the traditional approach as closely as possible by using the same model architecture, participant-wise 10-fold cross validation, the same data preparation process (with the test data for the global model imputed with the median of the withheld participants to retain restrictions on sharing patient data), and the same early stopping protocol (with rollback calculated with the MAE of the local training data). Each client used data from one participant and held out 20% of the participant’s data to evaluate the local model. The weights of the local models were aggregated using FedAvg (weighted mean of the models’ weights) to give the global model. This global model was then redistributed, and each client tailored the global model to their local participant by training on the participants’ training data with a lower learning rate (0.0001 compared to 0.001), this fine-tuned the model weights to each clients’ participant. These personalised versions of the global model were then evaluated using MAE, which was aggregated with a weighted average. The global model was then evaluated on the fold’s test participants’ data. This process was repeated for 100 rounds, all clients were used for every round, and the model weights were rolled back to the round with the lowest global MAE. This federated system was simulated with Flower v1.3.0 (Flower Framework, 2025) and on the same machine as the traditional approach.

### 2.6 Model evaluation

The performances of these models were evaluated using multiple metrics: MAE, Pearson rank correlation, intraclass correlations (ICC), joint plots, and Bland–Altman plots. The MAE was calculated using scikit-learn (Pedregosa et al., 2011) and the Pearson rank correlation was calculated using scipy (Virtanen et al., 2020). The ICC estimated the variability of different MDS-UPDRS-III scores of the same participant to the total variation across all scores and all participants. The average raters’ absolute ICC was calculated using pingouin (Vallat, 2018) (ICC3), where the participants were the targets, the MDS-UPDRS-III scores were the ratings, and the raters were the true MDS-UPDRS-III scores and the scores predicted by the model being evaluated. The joint plots were generated using seaborn (Waskom, 2021) and the Bland–Altman plots, which are shown in the Supplementary materials, using statsmodels (Seabold and Perktold, 2010).

## 3 Results

### 3.1 Data

89 PD participants were included in the following analyses. The majority of participants were male (*n* = 60, 67.4%) and a mean ± SD age of 69 ± 9 years at the first assessment (month 18). Table 1 shows the number of participants and samples (one sample per day) for the four visits, giving a total number of 1,476 samples.

**Table 1:** Number of participants and samples for each visit.

Visit (month)	18	36	54	72
Number of participants	47	64	57	49
Number of samples	303	431	404	338

**Figure 3:**
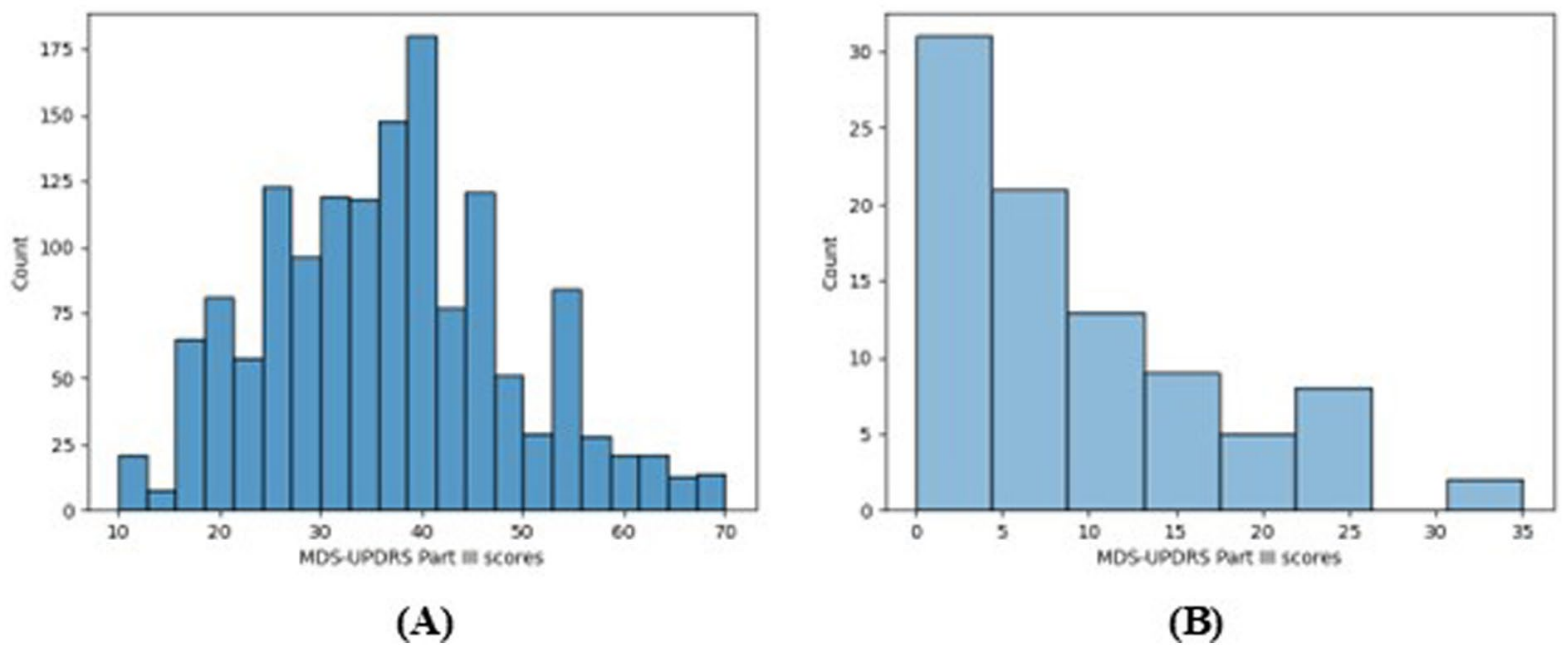
**(A)** Shows a histogram plot of the distribution of all MDS-UPDRS Part III scores in the data (up to four per participant) and **(B)** shows a histogram of the ranges of the participants’ MDS-UPDRS Part III scores.

The distribution of the participants’ MDS-UPDRS Part III total scores are shown in Figure 3. Figure 3A shows that there is a reasonably wide range of scores (10–70 from the available 0–132) and that the participants were living with relatively mild-to-moderate PD motor symptom severity. Figure 3B shows that most of the participants have clinically meaningful changes in their MDS-UPDRS Part III score over the 6-year study period. Therefore, these data are suitable for the analyses presented in this work.

### 3.2 Traditional machine learning

**Figure 4:**
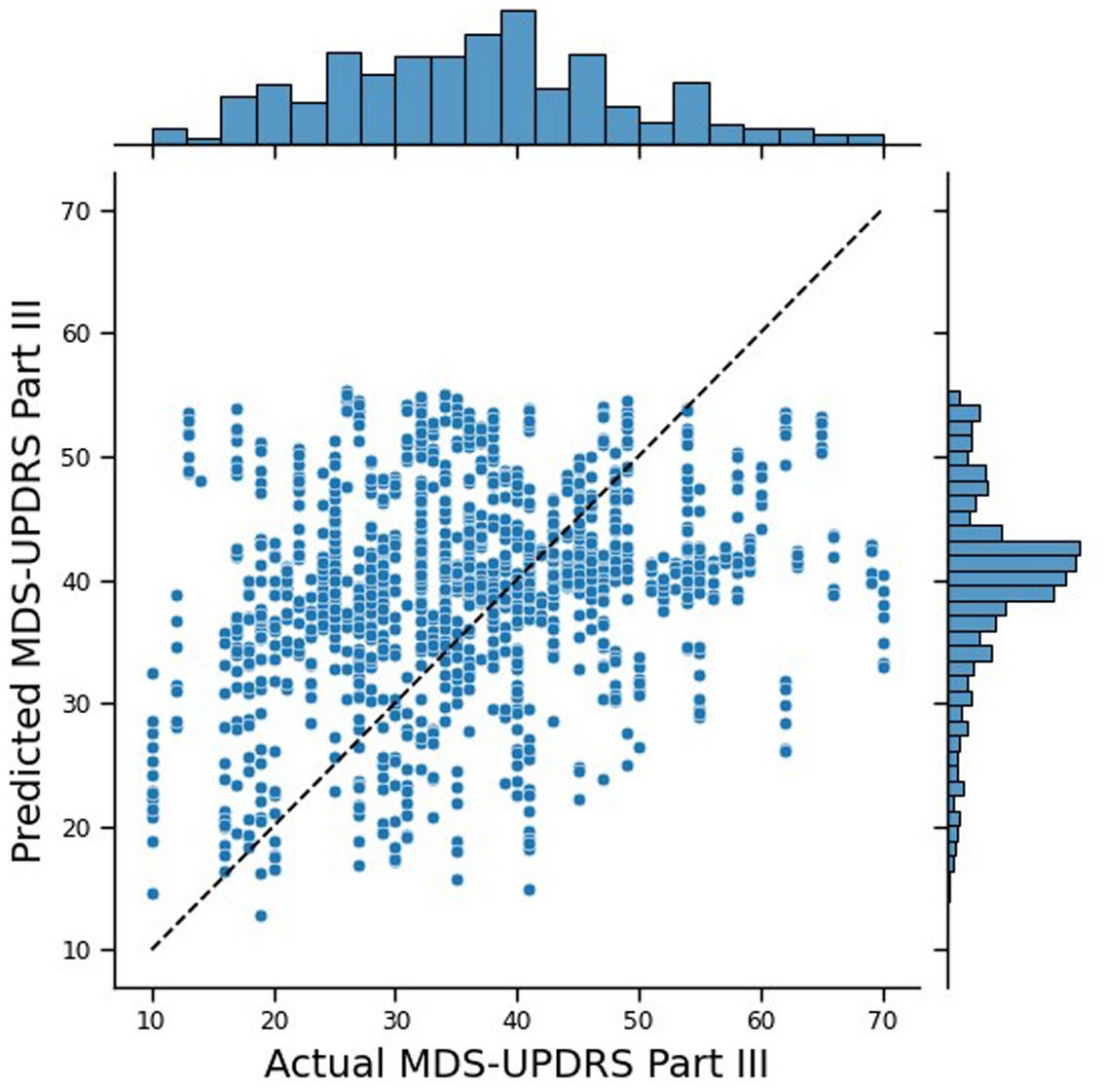
Joint plot of the MDS-UPDRS Part III scores predicted by the traditional NN, against the true MDS-UPDRS Part III scores. The black, dashed line represents a hypothetical perfect prediction.

The traditional Machine Learning approach performed reasonably well, with an MAE of 10.43. Figure 4 shows the joint plot of the predicted scores vs. the true scores. In addition, the Pearson rank correlation, r, of the predicted vs. true MDS-UPDRS III scores was 0.26 (*p* < 0.0001) and the ICC was 0.389 (*p* = 0.011). The distribution of the predicted scores shows a cluster around 40, tailing for lower scores, and no predictions over 56, whereas the true scores are more spread out and tailing for higher scores (up to 70). While this performance is insufficient for deployment for real-world estimation of MDS-UPDRS-III from digital wearables—likely due to the small number of participants in these data—these outcomes provide a good goal post for comparing the outcomes to that of a federated system.

### 3.3 Federated learning

**Figure 5:**
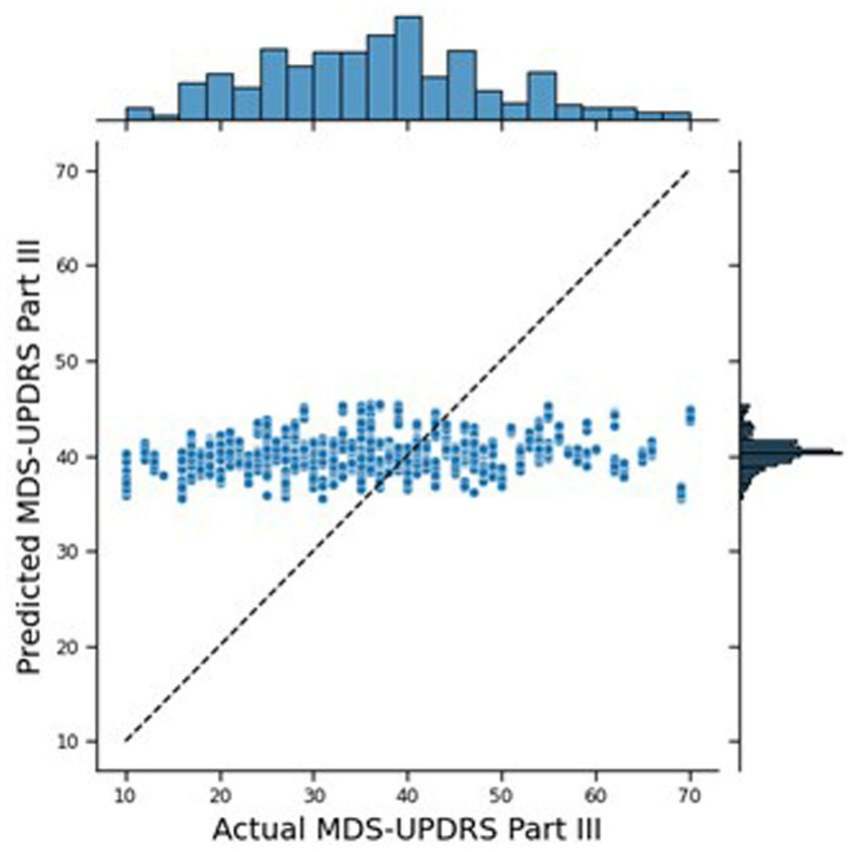
Joint plot of the MDS-UPDRS Part III scores predicted by the federated NN, against the true MDS-UPDRS Part III scores. The black, dashed line represents a hypothetical perfect prediction.

The global FL model had a MAE of 10.22 and the local, personalised models had an MAE (mean across the folds) of 4.83, demonstrating the effectiveness of personalising the global model to the local data. However, the correlation, r, was 0.17 (*p* < 0.0001) and the ICC was −0.05 (*p* = 0.59), indicating a substantial decrease in performance. The joint plot of the predicted scores vs. the true scores, Figure 5, shows underfitting of the global federated NN by only predicting values around 40, leading to the lower MAE. Therefore, the federated learning system still requires improvement to be comparable to the performance of the traditional model.

### 3.4 Feature importance

**Figure 6:**
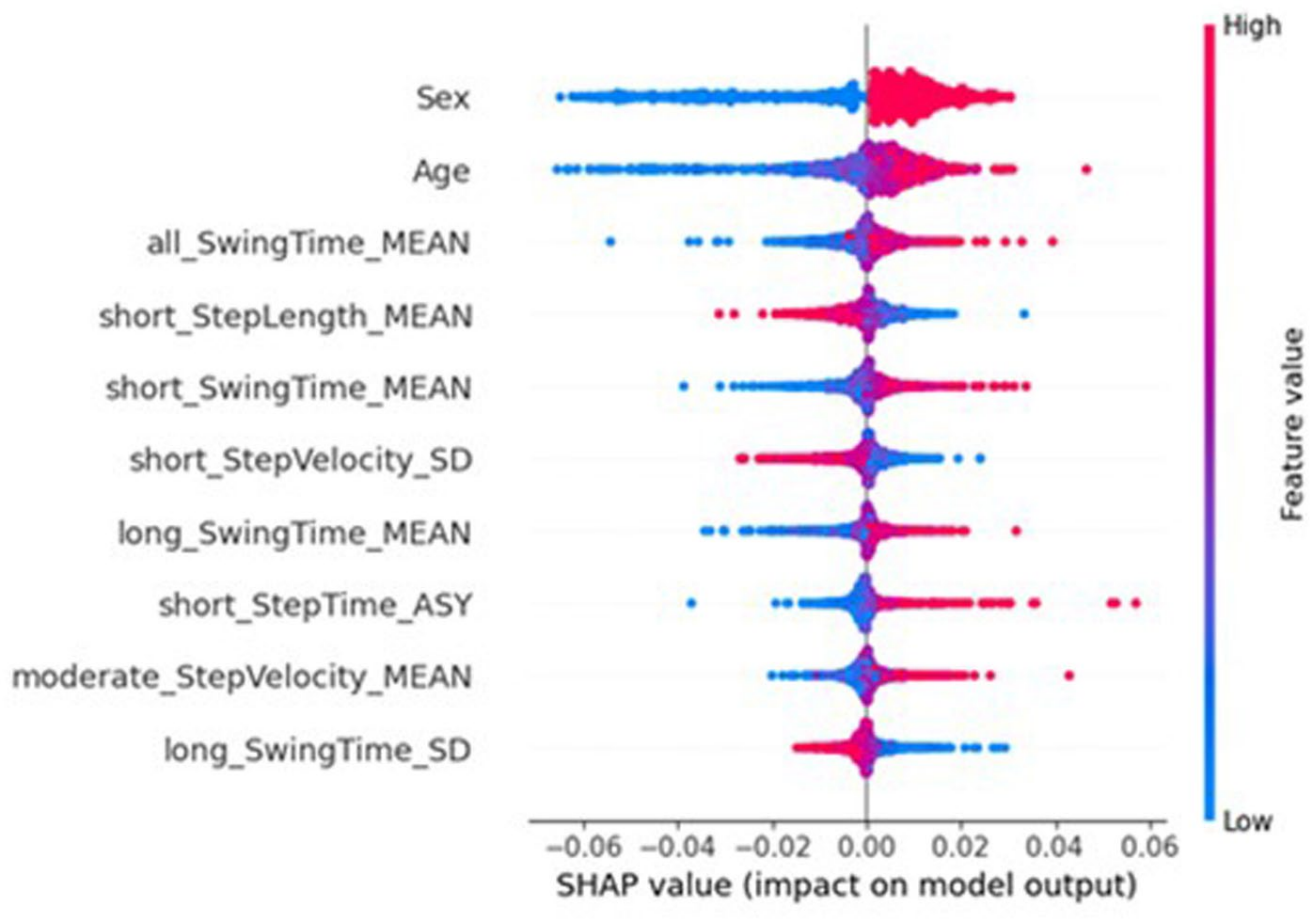
SHAP importances of the traditional NN. Showing the 10 most important features, with the more important feature higher up.

The underfitting of the global model may be due to the local models only being trained on data from one participant; the lack of representative data could be causing high bias in the local models, therefore extending to underfitting in the global model. To better understand the underlying mechanics of the models, and to explore the potential contributions to the federated learning system having a reduced performance, SHapley Additive exPlanations (SHAP) (Lundberg and Lee, 2017) [implemented with SHAP (Welcome to the SHAP documentation, 2025)] was used to investigate the importance of the features to the different models. SHAP is a method from game theory used to explain the output of a machine learning model by quantifying the contribution of each feature to each prediction (Welcome to the SHAP documentation, 2025; Marcilio and Eler, 2020). Figure 6 shows a beeswarm plot the SHAP values for the ten most important features for traditional NN. For each feature listed, each dot represents a sample, with its colour referring to the sample’s feature value, and its place on the x-axis referring to the sample’s impact on the model, i.e., a sample that lead to a large increase in the predicted MDS-UPDRS-III score would see the dot on the far right, and a small decrease in predicted MDS-UPDRS-III score would see the dot on the left, near the y-axis line. Figure 6 reports the ten most important features included two participant characteristics, sex and age, with mean absolute SHAP (MAS) values of 0.0161 and 0.0059. The sex of the participant was particularly effective, with females (blue) reducing the predicted scores, and males (red) increasing the predicted scores. Similarly, higher ages increased the predicted scores, whereas lower ages decreased predicted scores.

**Figure 7:**
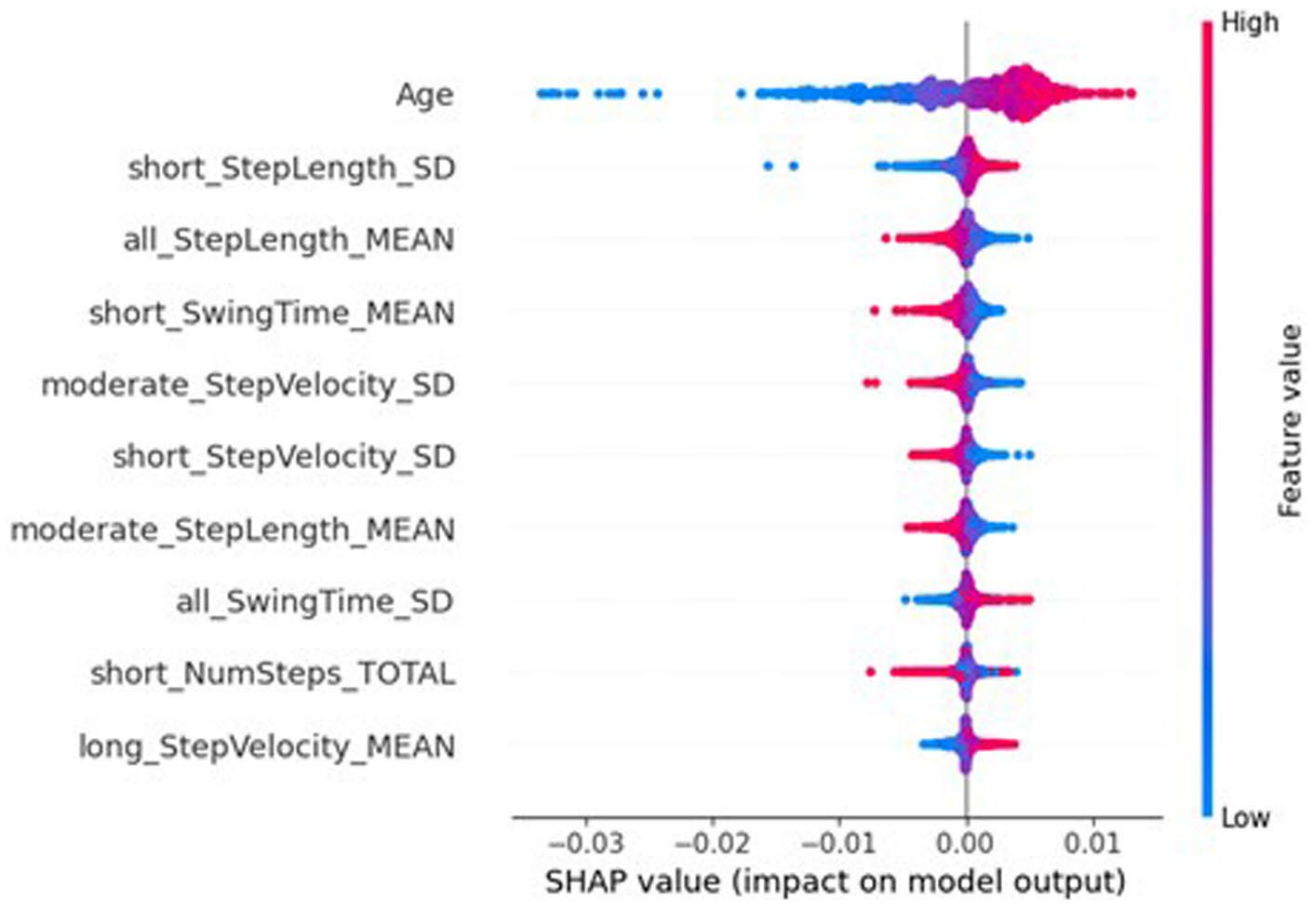
SHAP importances of the global model from the federated system. Showing the 10 most important features, with the more important features higher up.

**Figure 8:**
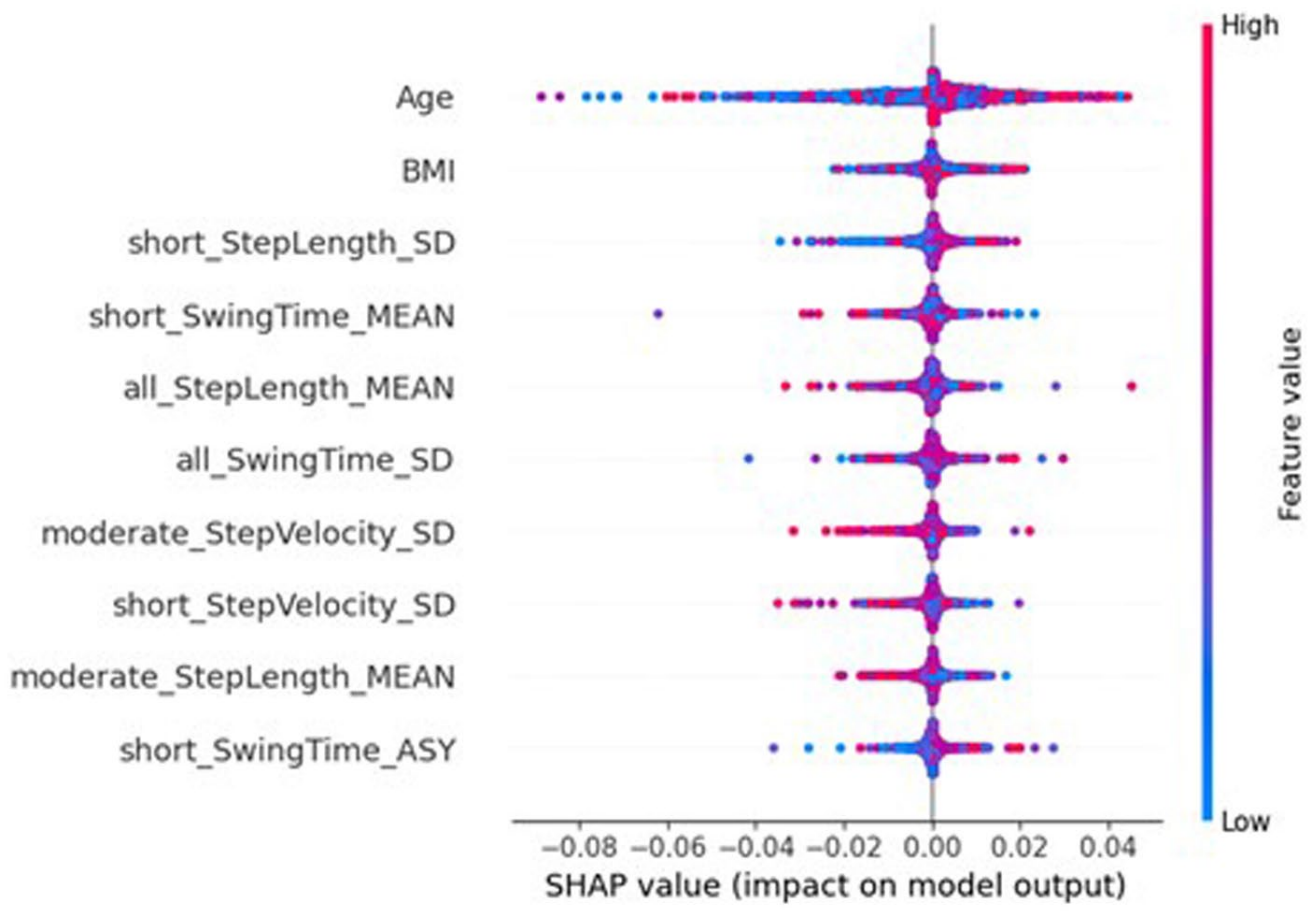
SHAP importances of the local models from the federated system. Showing the 10 most important features, with the more important features higher up.

However, the SHAP values of the global federated NN, top ten of which is shown in Figure 7, show the importance of participant’s sex dropped to 32nd place (MAS = 0.0003), leaving age as the most important (MAS = 0.0049), and the BMI increased from 32nd most important for the traditional NN (MAS = 0.0015) to 11th for the global federated NN (MAS = 0.0005). These changes can be better understood by examining the importances of the local, personalised models, shown in Figure 8, where age and BMI are the most important features (MAS = 0.0110 and 0.0027), but sex has a MAS of 0, with a SHAP value of 0 for every instance. Therefore, it appears that the lack of within-participant variation of the participant’s sex is leading to the local models “ignoring” this measure and therefore reducing the importance of this measure for the global federated NN. Due to the longitudinal nature of the ICICLE-GAIT study (conducted over 6 years), this problem does not extend to the participants’ age or BMI, but it will be present for clinical trials lasting only 6–12 months. This means that these participant characteristics, which are very important both clinically and to the traditional NN, are not being properly utilised in a federated system. This may be contributing to the underperformance seen in Figure 5. Additionally, the clear relationship that age had with the impact on the model output for the traditional centralised and the global federated models is lost for the local models. Indeed, it is uncertain whether higher feature values lead to an increase or decrease in the predicted value, for all measures shown in Figure 8. Therefore, through this analysis, we have highlighted key challenges in implementing Federated Learning in a smart-home scenario, or indeed any edge device FL case, for clinical studies.

## 4 Discussion

The current analysis has leveraged retrospective data and simulation analysis to examine the use of edge device federated learning to predict MDS-UPDRS Part III from gait measures derived from a lower-back accelerometer worn by PD participants. Importantly, federated learning showed a decrease in performance compared to the traditional machine learning approach, when inspecting the scatterplots of true vs. predicted scores. The three studies that have explored edge device FL for PD also saw, when compared to traditional ML, varied decreases in performance for FL: 81% balanced accuracy for traditional ML compared to 76% (Jorge et al., 2022) for FL, 87.23% accuracy compared to 86.98% (Soumma et al., 2024), and 73.5% balanced accuracy compared to 63.2% for their CNN and 72.1 to 66.2% for their CNN-LSTM (Jorge et al., 2024). Therefore, this decrease in performance is not only found in the current analysis.

This reduction in performance could be due to many factors, for example the number of training instances for the clients is substantially smaller than the number of the number of feature inputs, worsening a problem known as the “curse of dimensionality”. However, the current analysis has used XAI techniques to explore the underlining functionality of the NN to attempt to understand what may be contributing to these decreases in performance and highlighted a lack of within-participant variation in the data as a possible key factor. The following analyses explore potential solutions to this key challenge in deploying edge device FL for smart-home monitoring.

### 4.1 Potential solutions

#### 4.1.1 Server-side training

To attempt to resolve the lack of local variation in these participant characteristics for the clients, server-side training, as suggested by Ozdayi et al. (2020), was implemented to our smart-home scenario. This is a data-sharing technique and was done by reserving randomly selected 8% of training participants as “tuning data” for fine-tuning the global model on the central server after FedAvg aggregation. Similarly to the personalisation fine-tuning, a lower learning rate of 0.0001 was used.

**Figure 9:**
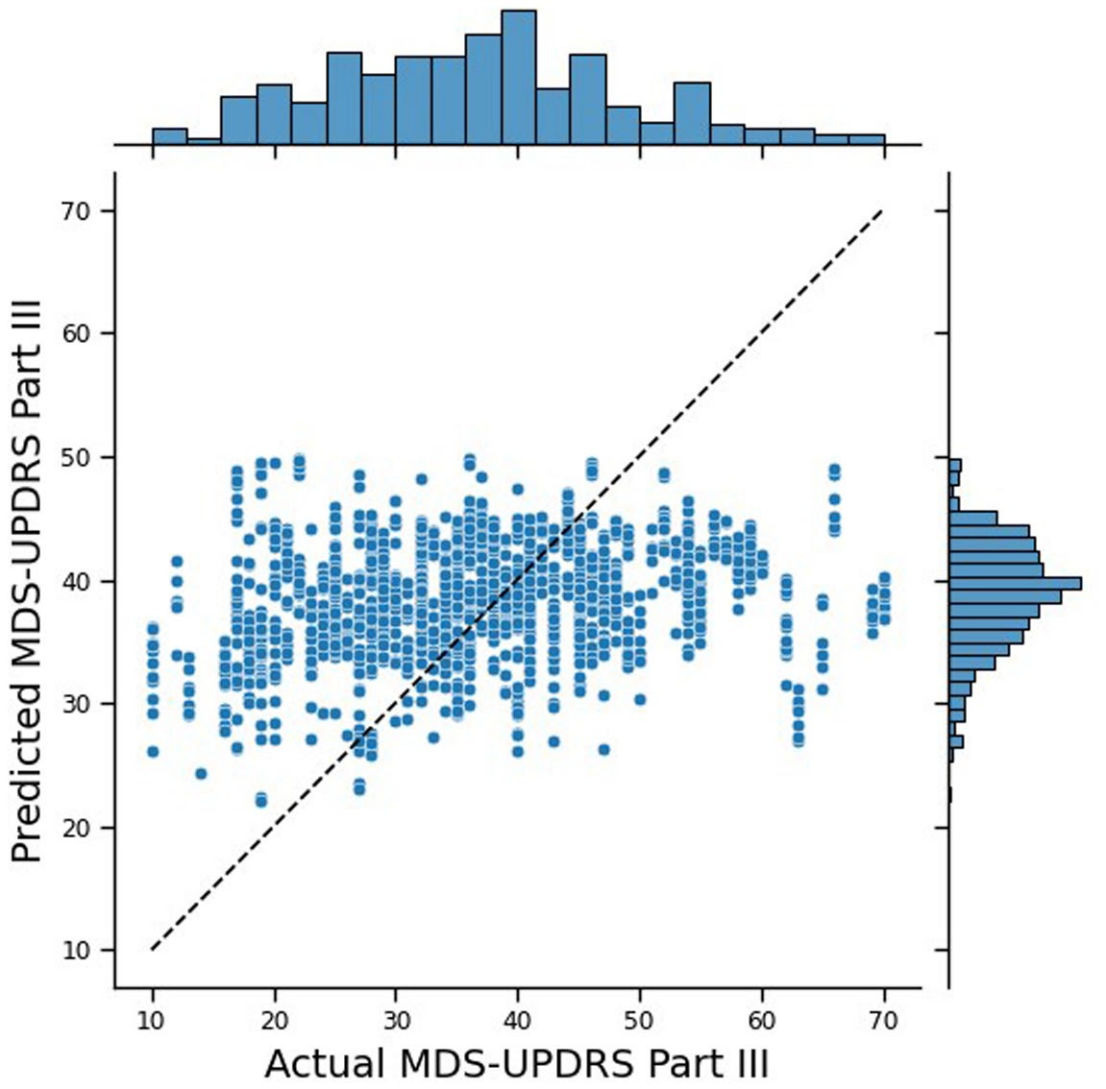
Joint plot of the MDS-UPDRS Part III scores predicted by the federated NN, with server-side training, against the true MDS-UPDRS Part III scores. The black, dashed line represents a hypothetical perfect prediction.

Using this server-side training improved the global model with an MAE of 9.81, r = 0.22 (*p* < 0.0001) and ICC was 0.318 (*p* = 0.037). Figure 9 shows the joint plot of the predicted scores vs. the true scores and demonstrates that this approach has improved the underfitting seen in the conventional FL. The local models had a mean MAE of 5.86, therefore this approach successfully improved the global model, but at a cost to local performance.

**Figure 10:**
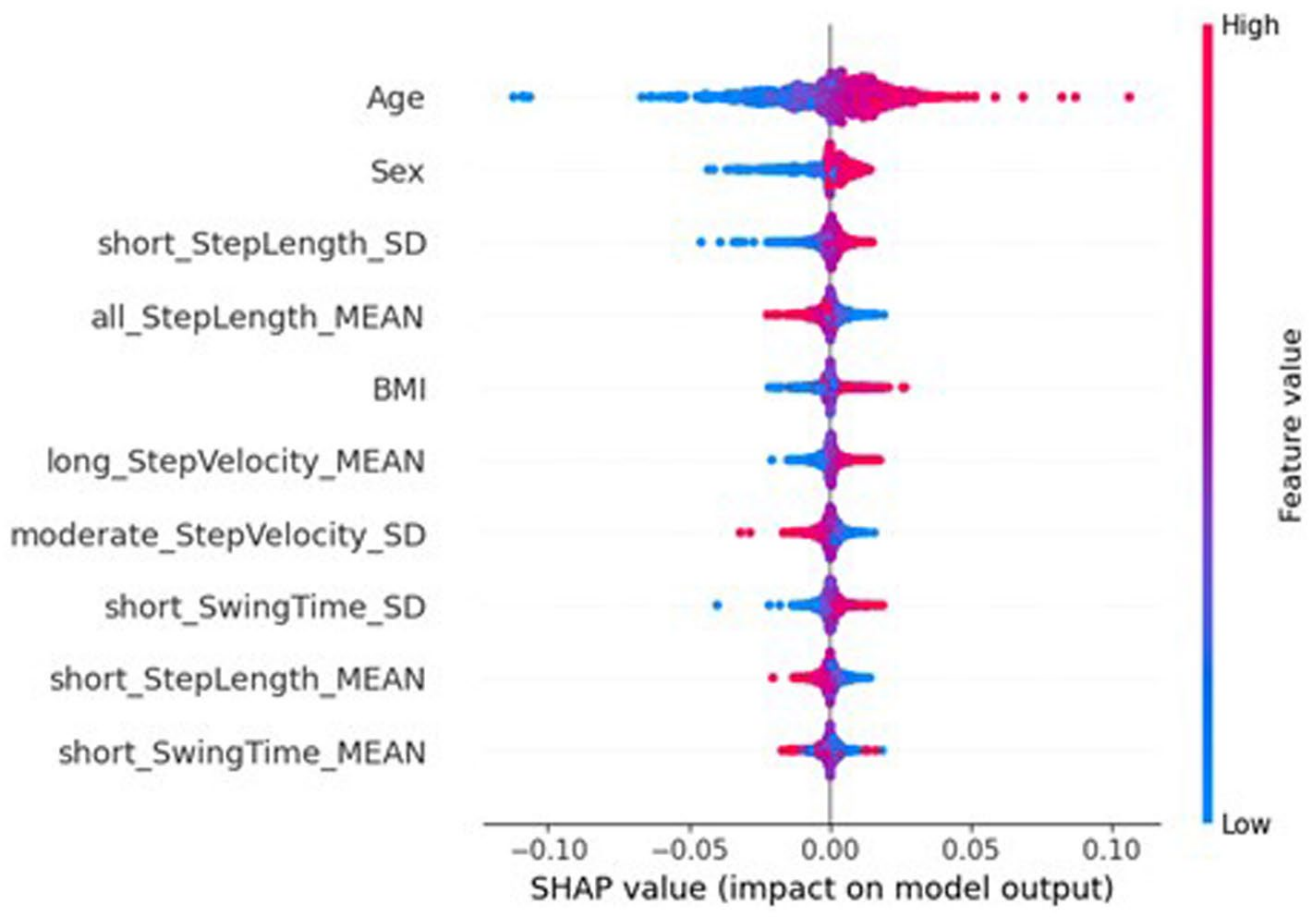
SHAP importances of the global model from the federated system, with server-side training. Showing the 10 most important features, with the more important feature higher up.

**Figure 11:**
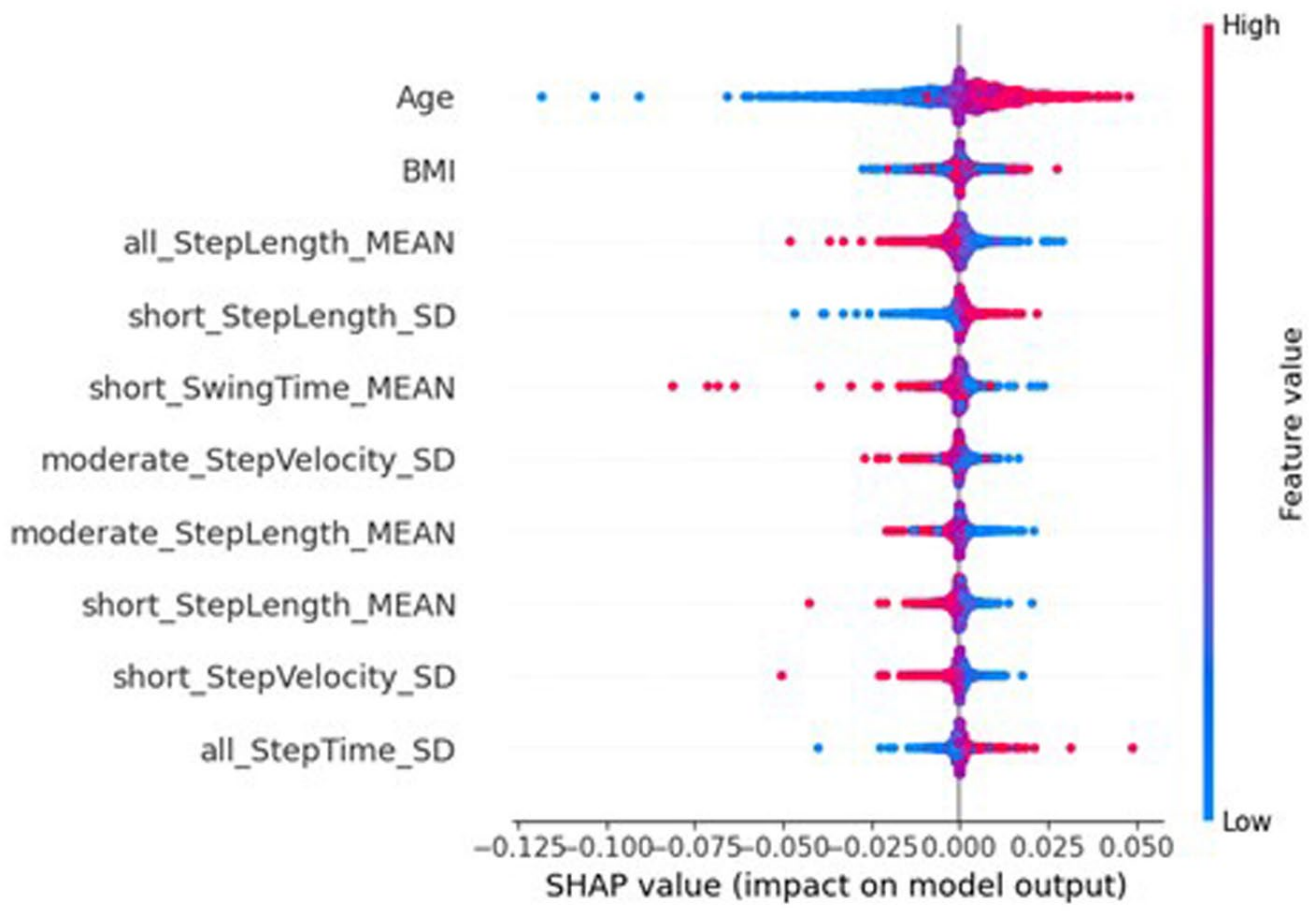
SHAP importances of the local models from the federated system, with server-side training. Showing the 10 most important features, with the more important feature higher up.

Figures 10, 11 show the SHAP importances of the global and local models, respectively, with this server-side training. Figure 10 shows that the importances of the global model with this approach better reflect the SHAP values of the traditional NN, with sex now the second most important feature, though the MAS value was reduced to 0.0056. Interestingly, the BMI has increased in importance ranking to 5th (MAS = 0.0026). However, this impact on the SHAP values for the global NN has not completely extended to the local models, where sex still has a SHAP value of 0 for every instance. Therefore, the personalisation process of fine-tuning the model on the local data is potentially causing this measure to be ignored by the local models. While Figure 11 shows minor variation in the order of the feature rankings with server-side training, this approach has improved the clarity and consistency of measures’ impacts on the model output, compared to conventional federated learning.

#### 4.1.2 Client-side data

We are also proposing an additional data sharing approach, “client-side data”. Here, the 8% withheld tuning participants from server-side training were instead added to the training data for the local models. When personalising the models, the samples from the client’s participant were weighted 4-to-1 to tune the model to the participant’s specific data without allowing the model to “forget” the importance of the participants’ characteristics. This 4:1 weighting ratio was selected based on experimentation ranging from 2:1 to 6:1 in a single fold. In this proposed scenario, these data would be stored on the devices and would not be transmitted at any point during deployment, preserving the privacy priorities of an FL system.

**Figure 12:**
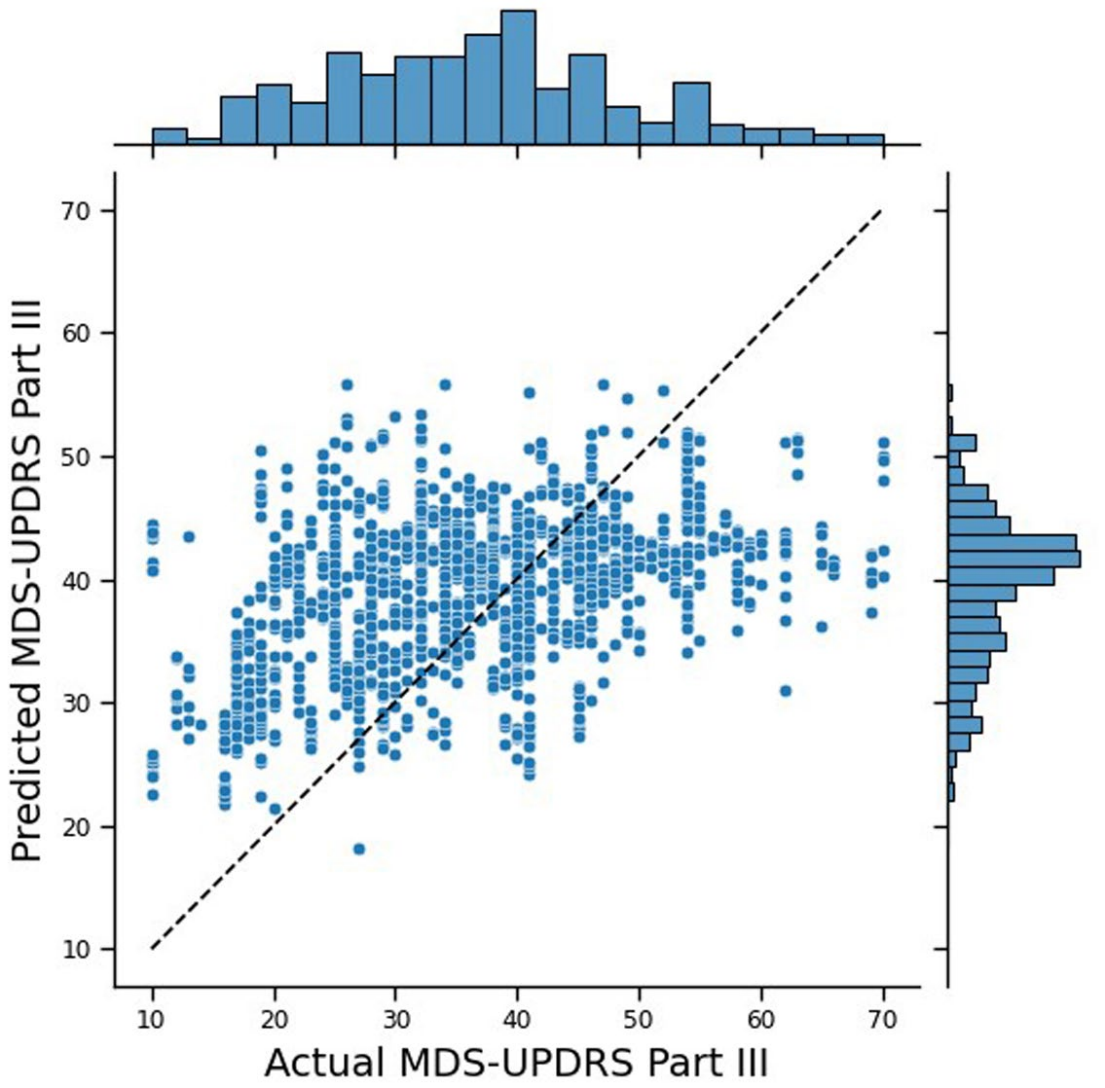
Joint plot of the MDS-UPDRS Part III scores predicted by the federated NN, with client-side data, against the true MDS-UPDRS Part III scores. The black, dashed line represents a hypothetical perfect prediction.

This approach produced the lowest MAE within this work, which was 9.26. The correlation and ICC were also improved and outperformed server-side training with r = 0.43 (*p* < 0.0001) and ICC = 0.438 (*p* = 0.04). Figure 12 shows that the predicted scores were the most similar to the true scores, beating the traditional centralised machine learning approach. However, despite weighting the samples when fine-tuning the local models, the MAE of the local models was 6.83, the highest of the federated approaches. Therefore, this improved global performance came at the cost of reduced local performance.

**Figure 13:**
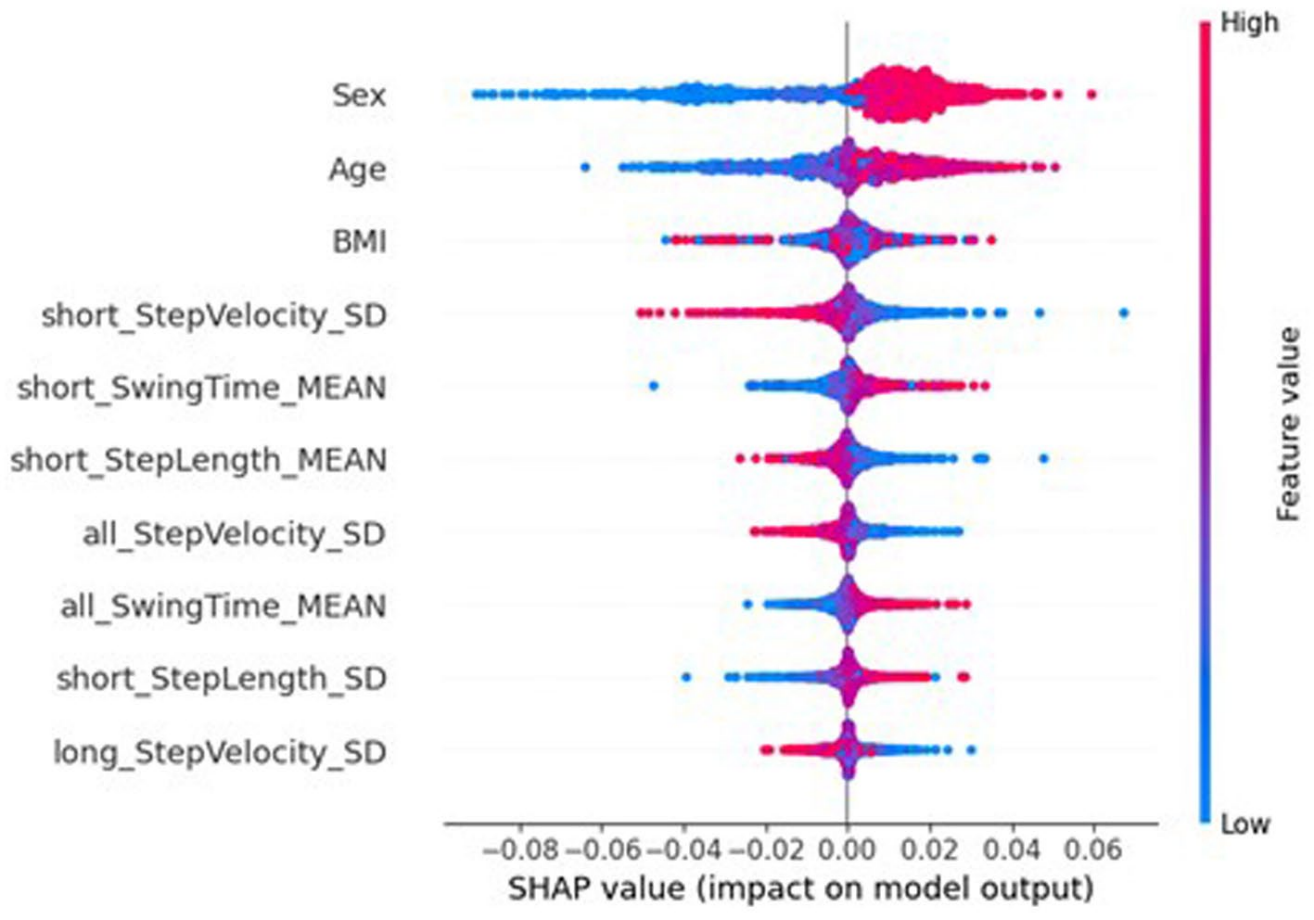
SHAP importances of the global model from the federated system, with client-side data. Showing the 10 most important features, with the more important feature higher up.

**Figure 14:**
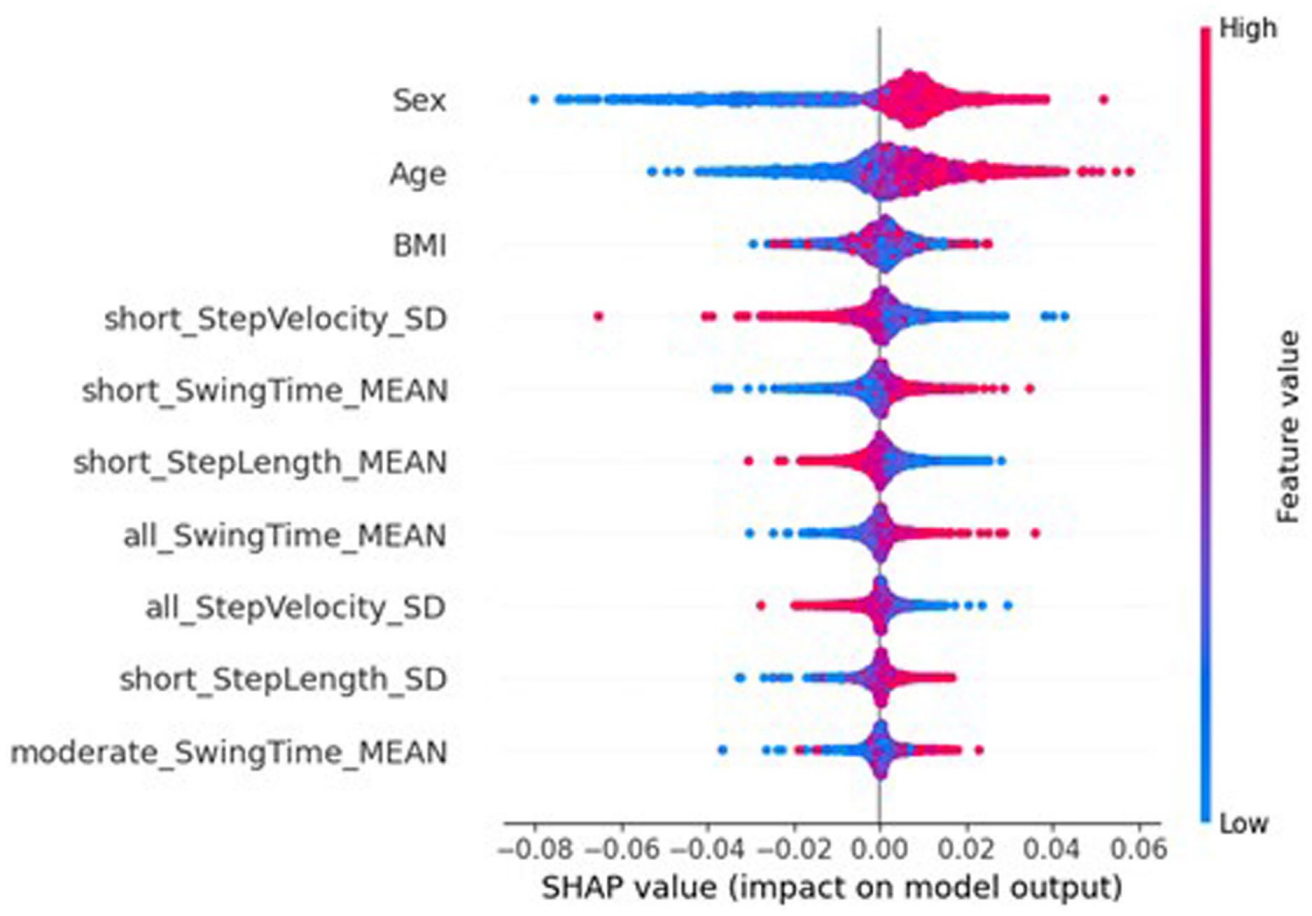
SHAP importances of the local models from the federated system, with client-side data. Showing the 10 most important features, with the more important feature higher up.

SHAP analysis of the global and local federated NN with client-side data are shown in Figures 13, 14, respectively. Sex, age, and BMI are the most important features for both the global and local models. Therefore, including some participants’ data successfully restored the participant characteristics as the most important features to the local models, which remained in the aggregation process for the global models. The top six most important features are the same for the local and global models, and the top nine are very similar (7th and 8th places have swapped), therefore there is more stability in the feature importances when using client-side data compared to the other FL approaches.

**Figure 15:**
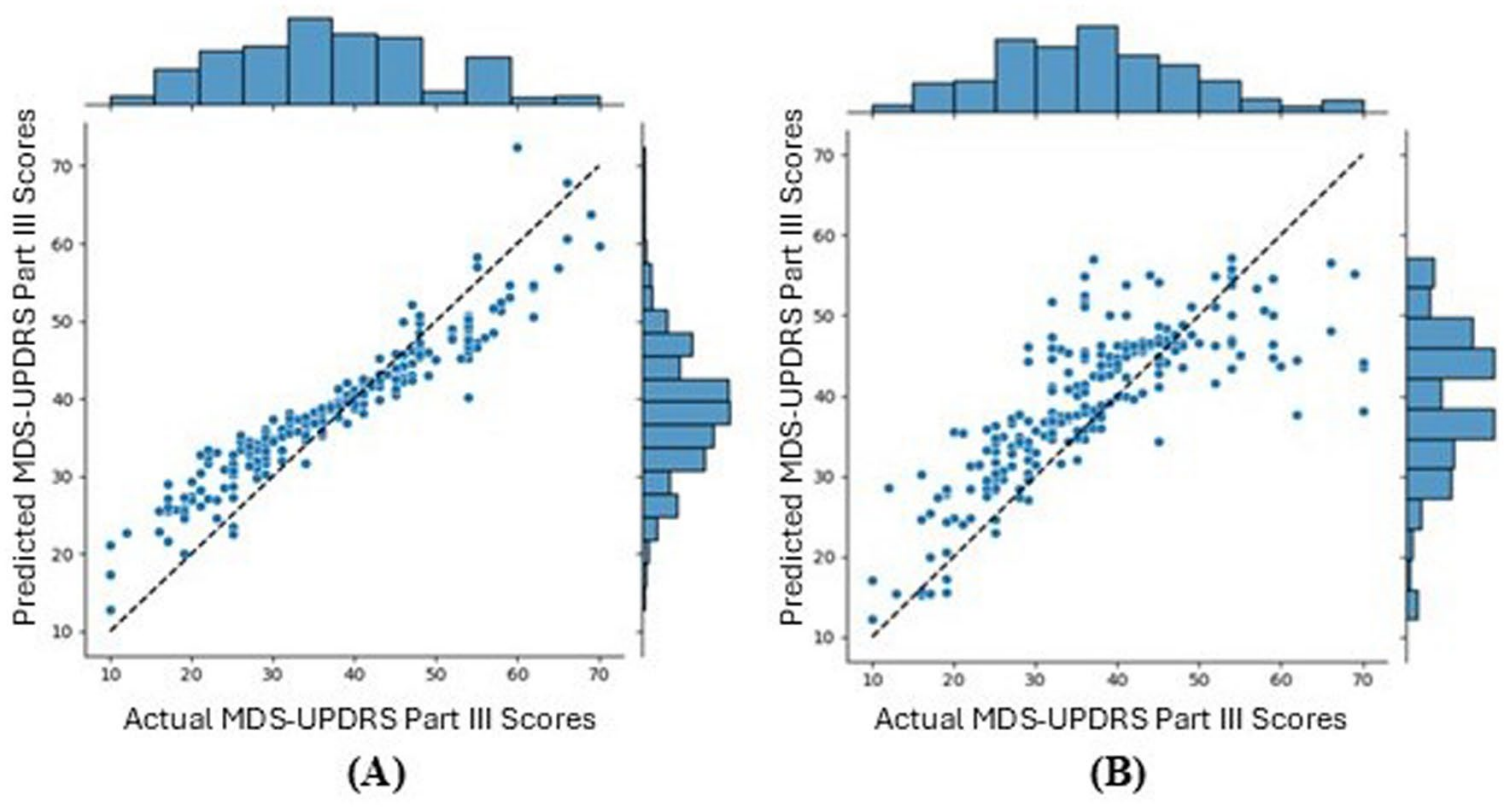
Joint plot of the MDS-UPDRS Part III scores predicted by the local models against the true MDS-UPDRS Part III scores, for the first fold. **(A)** Shows the conventional FL and **(B)** shows the FL with client-side data. The black, dashed line represents a hypothetical perfect prediction.

Client-side data improved the global model by reducing the MAE by 0.96, but increased the mean local MAE by 2.00. Unlike the conventional FL global model, the conventional FL local models were not underfit, as seen in Figure 15A, where the correlation r = 0.95 (p < 0.0001), highlighting the effectiveness of the model personalisation. Whereas, Figure 15B shows that for FL with client-side data, the local models had a reduced performance, with r = 0.79 (p < 0.0001) and underpredictions of higher true scores. Therefore, introducing extra patients’ data to the clients made the global model better for new patients but reduced performance for predicting new instances for participating patients. Therefore, it is possible that while the participant characteristics are helpful for predictions with unseen participants, as seen with the traditional NN, they may not be as helpful for within-participant predictions. Within the context of smart home technology for a clinical trial, this means that server-side training and client-side data may lead to a slightly reduced performance for participants within the study but would greatly improve the global model for any new participants. This is beneficial since in most cases, clinical trials do not run in perfect parallel with every participant starting and concluding at the same time.

### 4.2 Limitations

An important challenge with the current analysis is the use of pre-existing data that was not designed for this study. As such, there is a lack of labelled data since the MDS-UPDRS was only recorded once per visit, therefore the daily gait measures have only one score associated to all 7 days. This is a key limitation since daily changes in symptoms were not recorded, furthermore these symptoms will fluctuate throughout the day as dopaminergic medication is taken and wears off. Therefore, this lack of labelled data may contribute to the poor performance of both the traditional NN and the federated NNs presented in the current study. In addition, imputing missing values in test data from the test data median could possibly cause some data leakage, but was necessary to preserve the privacy goals of FL. Furthermore, the participants were all newly diagnosed and screened for cognitive impairment, meaning there is a substantial lack of more severe PD present in these data as well as a lack of very mild PD, as seen in Figure 1. Therefore, these models may not generalise well to participants living with PD that is at either extreme of severity.

Additionally, since local testing data were randomly selected from the patients’ available data, it is very likely that the same MDS-UPDRS-III score was present in both the local training and local testing data. Therefore, the local performance may have an unrealistic advantage. A limitation of the server-side training and client-side data approaches, where participants were withheld from the federated system, is that for real-world deployment it would require additional data and in this simulated analysis it reduces the number of clients that can contribute to FL training and local evaluation.

### 4.3 Future work

As a part of the TORUS project, we will collect data that is tailored to our specific research questions, which will be informed by our current experimentation. Future work will also explore the use of un-supervised or semi-supervised AI techniques that will be able to handle the lack of labelled data within this and similar datasets, since it is difficult to collect the MDS-UPDRS Part III score at frequent intervals.

Federated learning for smart homes presents an interesting opportunity to protect patients’ privacy during a clinical trial. While our analysis shows promise in simulated FL on retrospective data, there would be key challenges associated with deployment. For example, limited Wi-Fi speed and power outages in rural areas would present a challenge during model updates. Fortunately, FL can handle some missingness during model updates (these local models would not be included in the FedAvg process when creating the global model). Therefore, future work should explore the impacts of missing clients on FL training. Other challenges include the restricted access to raw patient data, which may be needed for validation in a clinical trial. In such cases, samples of raw data could be stored in the participants’ home until it can be collected (e.g., via courier). Thus, preserving the protection of patient data.

In addition, possible alternative approaches to improve FL performance include pretraining the local models on an alternate/public data set or masking the gait measures for a few rounds of model training so that the local models could learn the importance of these patient characteristics. Although, the models may “forget” these importances as model training progresses, meaning these additional steps would have minimal impact on the final outcomes. Alternatively, we could adjust the loss function – the function that calculates the difference between the predicted and true scores – to enforce the local model weights to reflect those in the traditional NN. However, many of these suggestions would require additional data and may not translate well to similar data (i.e., other disease cohorts). An ideal solution would be a federated aggregation method that is able to allow the global model to identify the importance of participant characteristics, despite their lack of variation locally, possible solutions could include clustering the clients, meta-learning, or multi-task learning (Karami and Karami, 2025).

## 5 Conclusion

In this paper, we have explored the efficacy of FL to predict PD symptom severity from real-world measures of mobility through simulated analysis and highlighted key challenges of edge device FL with medical data. We have explored outcomes of both local and global models and attempted to understand the impact of FL on model weights with XAI techniques. While the global FL NN slightly outperformed the traditional NN, with MAEs of 10.22 compared to 10.42, the global performance was underfit and was improved by using server-side training (MAE = 9.81) and client-side data (MAE = 9.26) when training the system. These additional FL approaches successfully restored the importance of participant characteristics, such as sex, that were lost by the conventional FL system due to a lack of variation in the local data. However, these global improvements came at the cost of reduced local performances, with MAEs of 4.83, 5.86, and 6.83 for the conventional FL, server-side training, and client-side data approaches. While more experimentation is required, this work has shown that FL holds promise for a smart-home system that prioritises privacy and personalisation for its users.

## Data Availability

The data analyzed in this study is subject to the following licenses/restrictions: the data for this study can be made available upon request. Requests to access these datasets should be directed to Lisa Alcock, lisa.alcock@newcastle.ac.uk.
