# Supplementary material for: Privacy and personalisation: predicting Parkinson’s disease severity from real-world gait with federated learning
Source: Front Aging Neurosci. 2026 Mar 9;18:1766599. doi: 10.3389/fnagi.2026.1766599 (PMC13006630; doi:10.3389/fnagi.2026.1766599)
Supplement: Supplementary file 1 [file Data_Sheet_1.docx]

Supplementary Material

# Bland-Altman Plots

**
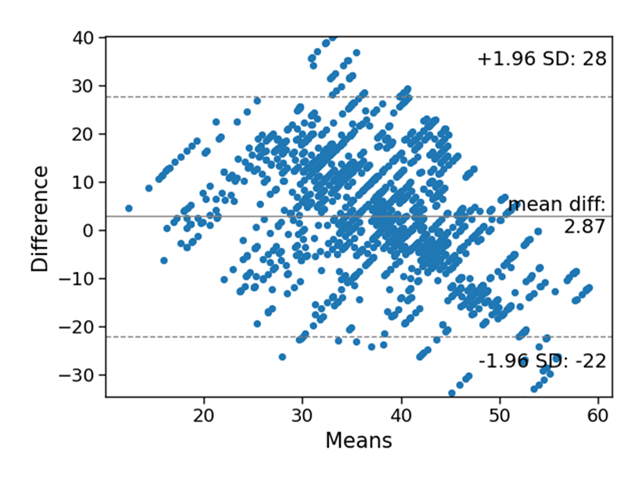
**

**Supplementary Figure 1.** Bland-Altman plot of the MDS-UPDRS Part III scores predicted by the traditional NN, against the true MDS-UPDRS Part III scores.

**
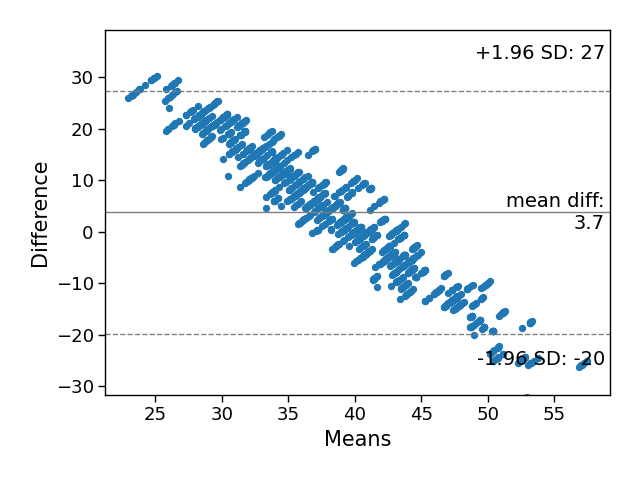
**

**Supplementary Figure 2.** Bland-Altman plot of the MDS-UPDRS Part III scores predicted by the federated NN, against the true MDS-UPDRS Part III scores.

**
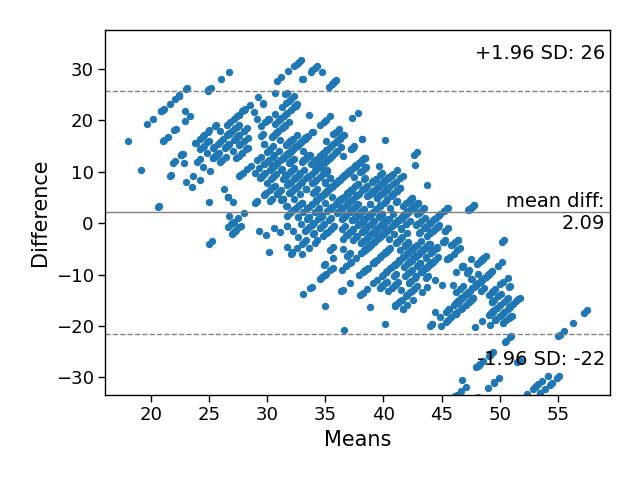
**

**Supplementary Figure 3.** Bland-Altman plot of the MDS-UPDRS Part III scores predicted by the federated NN, with server-side training, against the true MDS-UPDRS Part III scores.

**
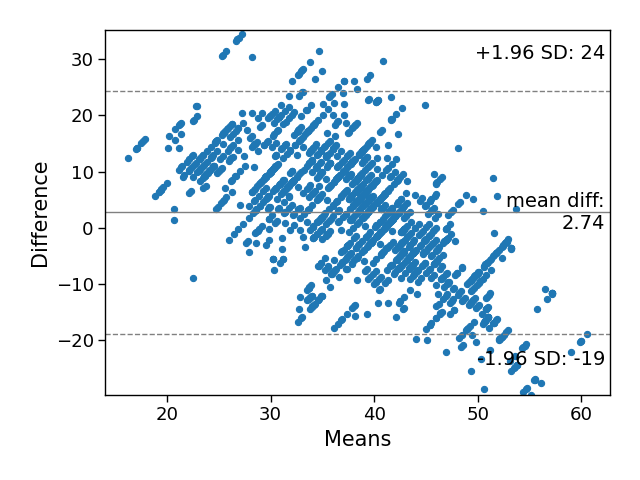
**

**Supplementary Figure 4.** Bland-Altman plot of the MDS-UPDRS Part III scores predicted by the federated NN, with client-side data, against the true MDS-UPDRS Part III scores.
